# The impossibility of low-rank representations for triangle-rich complex networks

**DOI:** 10.1073/pnas.1911030117

**Published:** 2020-03-02

**Authors:** C. Seshadhri, Aneesh Sharma, Andrew Stolman, Ashish Goel

**Affiliations:** ^a^Department of Computer Science, University of California, Santa Cruz, CA 95064;; ^b^Google, Mountain View, CA 94043;; ^c^Department of Management Science and Engineering, Stanford University, Stanford, CA 94305

**Keywords:** graph embeddings, graph representations, low-dimensional embeddings, low-rank representations, singular value decomposition

## Abstract

The study of complex networks is a significant development in modern science, and has enriched the social sciences, biology, physics, and computer science. Models and algorithms for such networks are pervasive in our society, and impact human behavior via social networks, search engines, and recommender systems, to name a few. A widely used algorithmic technique for modeling such complex networks is to construct a low-dimensional Euclidean embedding of the vertices of the network, where proximity of vertices is interpreted as the likelihood of an edge. Contrary to the common view, we argue that such graph embeddings do not capture salient properties of complex networks. The two properties we focus on are low degree and large clustering coefficients, which have been widely established to be empirically true for real-world networks. We mathematically prove that any embedding (that uses dot products to measure similarity) that can successfully create these two properties must have a rank that is nearly linear in the number of vertices. Among other implications, this establishes that popular embedding techniques such as singular value decomposition and node2vec fail to capture significant structural aspects of real-world complex networks. Furthermore, we empirically study a number of different embedding techniques based on dot product, and show that they all fail to capture the triangle structure.

## Significance

Our main message is that the popular method of low-dimensional embeddings provably cannot capture important properties of real-world complex networks. A widely used algorithmic technique for modeling these networks is to construct a low-dimensional Euclidean embedding of the vertices of the network, where proximity of vertices is interpreted as the likelihood of an edge. Contrary to common wisdom, we argue that such graph embeddings do not capture salient properties of complex networks. We mathematically prove that low-dimensional embeddings cannot generate graphs with both low average degree and large clustering coefficients, which have been widely established to be empirically true for real-world networks. This establishes that popular low-dimensional embedding methods fail to capture significant structural aspects of real-world complex networks.

Complex networks (or graphs) are a fundamental object of study in modern science, across domains as diverse as the social sciences, biology, physics, computer science, and engineering (1–3). Designing good models for these networks is a crucial area of research, and also affects society at large, given the role of online social networks in modern human interaction (4–6). Complex networks are massive, high-dimensional, discrete objects, and are challenging to work with in a modeling context. A common method of dealing with this challenge is to construct a low-dimensional Euclidean embedding that tries to capture the structure of the network (see ref. 7 for a recent survey). Formally, we think of the n vertices as vectors ${\vec{v}}_{1},{\vec{v}}_{2},\ldots ,{\vec{v}}_{n}\in R^{d}$, where d is typically constant (or very slowly growing in n). The likelihood of an edge (i,j) is proportional to (usually a nonnegative monotone function in) ${\vec{v}}_{i}\cdot {\vec{v}}_{j}$ (8, 9). This gives a graph distribution that the observed network is assumed to be generated from.

The most important method to get such embeddings is the singular value decomposition (SVD) or other matrix factorizations of the adjacency matrix (8). Recently, there has also been an explosion of interest in using methods from deep neural networks to learn such graph embeddings (9–12) (refer to ref. 7 for more references). Regardless of the specific method, a key goal in building an embedding is to keep the dimension d small—while trying to preserve the network structure—as the embeddings are used in a variety of downstream modeling tasks such as graph clustering, nearest-neighbor search, and link prediction (13). Yet a fundamental question remains unanswered: To what extent do such low-dimensional embeddings actually capture the structure of a complex network?

These models are often justified by treating the (few) dimensions as “interests” of individuals, and using similarity of interests (dot product) to form edges. Contrary to the dominant view, we argue that low-dimensional embeddings are not good representations of complex networks. We demonstrate mathematically and empirically that they lose local structure, one of the hallmarks of complex networks. This runs counter to the ubiquitous use of SVD in data analysis. The weaknesses of SVD have been empirically observed in recommendation tasks (14–16), and our result provides a mathematical validation of these findings.

Let us define the setting formally. Consider a set of vectors ${\vec{v}}_{1},{\vec{v}}_{2},\ldots ,{\vec{v}}_{n}\in R^{d}$ (denoted by the d×n matrix V) used to represent the n vertices in a network. Let $G_{V}$ denote the following distribution of graphs over the vertex set [n]. For each index pair i,j, independently insert (undirected) edge (i,j) with probability $\max\left(0,\min\left({\vec{v}}_{i}\cdot {\vec{v}}_{j},1\right)\right)$. (If ${\vec{v}}_{i}\cdot {\vec{v}}_{j}$ is negative, (i,j) is never inserted. If ${\vec{v}}_{i}\cdot {\vec{v}}_{j}\geq 1$, (i,j) is always inserted.) We will refer to this model as the “embedding” of a graph G, and focus on this formulation in our theoretical results. This is a standard model in the literature, and subsumes the classic Stochastic Block Model (17) and Random Dot Product Model (18, 19). There are alternate models that use different functions of the dot product for the edge probability, which are discussed in *Alternate Models*. Matrix factorization is a popular method to obtain such a vector representation: The original adjacency matrix A is “factorized” as $V^{T}V$, where the columns of V are ${\vec{v}}_{1},{\vec{v}}_{2},\ldots ,{\vec{v}}_{n}$.

Two hallmarks of real-world graphs are 1) sparsity, where the average degree is typically constant with respect to n, and 2) triangle density, where there are many triangles incident to low-degree vertices (5, 20–22). The large number of triangles is considered a local manifestation of community structure. Triangle counts have a rich history in the analysis and algorithmics of complex networks. Concretely, we measure these properties simultaneously as follows.

**Definition 1.** *For parameters* c>1 *and* Δ>0*, a graph* G *with* n *vertices has a* (c,Δ)*-triangle foundation if there are at least* Δn *triangles contained among vertices of degree, at most,* c*. Formally, let* $S_{c}$ *be the set of vertices of degree, at most,* c*. Then, the number of triangles in the graph induced by* $S_{c}$ *is at least* Δn.

**Fig. 1.**
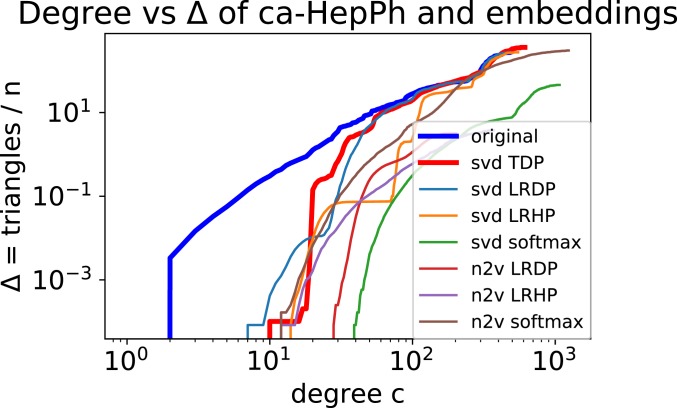
Plots of degree c vs. Δ: For a High Energy Physics coauthorship network, we plot c versus the total number of triangles only involving vertices of degree, at most, c. We divide the latter by the total number of vertices n, so it corresponds to Δ, as in **Definition 1**. We plot these both for the original graph (in thick blue) and for a variety of embeddings (explained in *Alternate Models*). For each embedding, we plot the maximum Δ in a set of 100 samples from a 100-dimensional embedding. The embedding analyzed by our main theorem (TDP) is given in thick red. Observe how the embeddings generate graphs with very few triangles among low-degree vertices. The gap in Δ for low degree is two to three orders of magnitude. The other lines correspond to alternate embeddings, using the node2vec vectors and/or different functions of the dot product.

Typically, we think of both c and Δ as constants. We emphasize that n is the total number of vertices in G, not the number of vertices in S (as defined above). Refer to real-world graphs in Table 1. In Fig. 1, we plot the value of c vs. Δ. (Specifically, the y axis is the number of triangles divided by n.) This is obtained by simply counting the number of triangles contained in the set of vertices of degree, at most, c. Observe that, for all graphs, for c∈[10,50], we get a value of Δ>1 (in many cases, Δ>10).

**Table 1.** Datasets used

Dataset name	Network type	Number of nodes	Number of edges
Facebook (29)	Social network	4,000	88,000
cit-HePh (31, 32)	Citation	34,000	420,000
String_hs (30)	PPI	19,000	5.6 million
ca-HepPh (29)	Coauthorship	12,000	120 million

All numbers are rounded to one decimal point of precision. PPI, protein–protein interaction.

Our main result is that any embedding of graphs that generates graphs with (c,Δ)-triangle foundations, with constant c,Δ, must have near-linear rank. This contradicts the belief that low-dimensional embeddings capture the structure of real-world complex networks.

**Theorem 1.** *Fix* c>4,Δ>0*. Suppose the expected number of triangles in* $G\approx G_{V}$ *that only involve vertices of expected degree* c *is at least* Δn*. Then, the rank of* V *is at least* $\min\left(1,poly\left(\Delta /c\right)\right)n/{\mathrm{lg}}^{2}n$.

Equivalently, graphs generated from low-dimensional embeddings cannot contain many triangles only on low-degree vertices. We point out an important implication of this theorem for Stochastic Block Models. In this model, each vertex is modeled as a vector in ${\left[0,1\right]}^{d}$, where the ith entry indicates the likelihood of being in the ith community. The probability of an edge is exactly the dot product. In community detection applications, d is thought of as a constant, or at least as much smaller than n. On the contrary, **Theorem 1** implies that d must be $\Omega \left(n/{\mathrm{lg}}^{2}n\right)$ to accurately model the low-degree triangle behavior.

## Empirical Validation

We empirically validate the theory on a collection of complex networks detailed in Table 1. For each real-world graph, we compute a 100-dimensional embedding through SVD (basically, the top 100 singular vectors of the adjacency matrix). We generate 100 samples of graphs from these embeddings, and compute their c vs. Δ plot. This is plotted with the true c vs. Δ plot. (To account for statistical variation, we plot the maximum value of Δ observed in the samples, over all graphs. The variation observed was negligible.) Fig. 1 shows such a plot for a physics coauthorship network. More results are given in *SI Appendix*.

Note that this plot is significantly off the mark at low degrees for the embedding. Around the lowest degree, the value of Δ (for the graphs generated by the embedding) is two to three order of magnitude smaller than the original value. This demonstrates that the local triangle structure is destroyed around low-degree vertices. Interestingly, the total number of triangles is preserved well, as shown toward the right side of each plot. Thus, a nuanced view of the triangle distribution, as given in **Definition 1**, is required to see the shortcomings of low dimensional embeddings.

## Alternate Models

We note that several other functions of dot product have been proposed in the literature, such as the softmax function (10, 12) and linear models of the dot product (7). **Theorem 1** does not have direct implications for such models, but our empirical validation holds for them as well. The embedding in **Theorem 1** uses the *truncated dot product* (TDP) function $\max\left(0,\min\left({\vec{v}}_{i}\cdot {\vec{v}}_{j},1\right)\right)$ to model edge probabilities. We construct other embeddings that compute edge probabilities using machine learning models with the dot product and Hadamard product as features. This subsumes linear models as given in ref. 7. Indeed, the TDP can be smoothly approximated as a logistic function. We also consider (scaled) softmax functions, as in ref. 10, and standard machine learning models [Logistic Regression on the Dot Product (LRDP) and Logistic Regression on the Hadamard Product (LRHP)]. (Details about these models are given in *Alternate Graph Models*.)

For each of these models (softmax, LRDP, and LRHP), we perform the same experiment described above. Fig. 1 also shows the plots for these other models. Observe that none of them capture the low-degree triangle structure, and their Δ values are all two to three orders of magnitude lower than the original.

In addition (to the extent possible), we compute vector embeddings from a recent deep learning-based method [node2vec (12)]. We again use all of the edge probability models discussed above, and perform an identical experiment (in Fig. 1, these are denoted by “n2v”). Again, we observe that the low-degree triangle behavior is not captured by these deep learned embeddings.

## Broader Context

The use of geometric embeddings for graph analysis has a rich history, arguably going back to spectral clustering (23). In recent years, the Stochastic Block Model has become quite popular in the statistics and algorithms community (17), and the Random Dot Product Graph model is a generalization of this notion [refer to recent surveys (19, 24)]. As mentioned earlier, **Theorem 1** brings into question the standard uses of these methods to model social networks. The use of vectors to represent vertices is sometimes referred to as *latent space models*, where geometric proximity models the likelihood of an edge. Although dot products are widely used, we note that some classic latent space approaches use Euclidean distance (as opposed to dot product) to model edge probabilities (25), and this may avoid the lower bound of **Theorem 1**. Beyond graph analysis, the method of Latent Semantic Indexing also falls in the setting of **Theorem 1**, wherein we have a low-dimensional embedding of “objects” (like documents), and similarity is measured by dot product (https://en.wikipedia.org/wiki/Latent_semantic_analysis).

## High-Level Description of the Proof

In this section, we sketch the proof of **Theorem 1**. The sketch provides sufficient detail for a reader who wants to understand the reasoning behind our result, but is not concerned with technical details. We will make the simplifying assumption that all $v_{i}$ have the same length L. We note that this setting is interesting in its own right, since it is often the case, in practice, that all vectors are nonnegative and normalized. In this case, we get a stronger rank lower bound that is linear in n. *Dealing with Varying Lengths* provides intuition on how we can remove this assumption. The full details of the proof are given in *Proof of Theorem 1*.

First, we lower-bound L. By Cauchy–Schwartz, ${\vec{v}}_{i}\cdot {\vec{v}}_{j}\leq L^{2}$. Let $X_{i,j}$ be the indicator random variable for the edge (i,j) being present. Observe that all $X_{i,j}$ are independent, and $\text{E}\left[X_{i,j}\right]=\min\left({\vec{v}}_{i}\cdot {\vec{v}}_{j},1\right)\leq L^{2}$.

The expected number of triangles in $G\approx G_{V}$ is

$$\text{E}\left[\underset{i\neq j\neq k}{\sum }X_{i,j}X_{j,k}X_{i,k}\right]$$ [1]

$$\leq \underset{i}{\sum }\underset{j,k}{\sum }\text{E}\left[X_{j,k}\right]\text{E}\left[X_{i,j}\right]\text{E}\left[X_{i,k}\right]$$ [2]

$$\leq L^{2}\underset{i}{\sum }\underset{j,k}{\sum }\text{E}\left[X_{i,j}\right]\text{E}\left[X_{i,k}\right]=L^{2}\underset{i}{\sum }{\left(\underset{j}{\sum }\text{E}\left[X_{i,j}\right]\right)}^{2}.$$ [3]

Note that $\sum_{j}\text{E}\left[X_{i,j}\right]=\text{E}\left[\sum_{j}X_{i,j}\right]$ is, at most, the degree of i, which is, at most, c. (Technically, the $X_{i,i}$ term creates a self-loop, so the correct upper bound is c+1. For the sake of cleaner expressions, we omit the additive +1 in this sketch.)

The expected number of triangles is at least Δn. Plugging these bounds in,

$$\Delta n\leq L^{2}c^{2}n\Rightarrow L\geq \sqrt{\Delta }/c.$$ [4]

Thus, the vectors have a length of at least $\sqrt{\Delta }/c$. Now, we lower-bound the rank of V. It will be convenient to deal with the Gram matrix $M=V^{T}V$, which has the same rank as V. Observe that $M_{i,j}={\vec{v}}_{i}\cdot {\vec{v}}_{j}\leq L^{2}$. We will use the following lemma stated first by Swanapoel (26), but which has appeared in numerous forms previously

**Lemma 1** (Rank lemma). *Consider any square matrix* $M\in R^{n\times n}$*. Then*

$$rank\left(M\right)\geq \frac{{\left|\underset{i}{\sum }M_{i,i}\right|}^{2}}{\left(\underset{i}{\sum }\underset{j}{\sum }{\left|M_{i,j}\right|}^{2}\right)}.$$

Note that $M_{i,i}={\vec{v}}_{i}\cdot {\vec{v}}_{i}=L^{2}$, so the numerator ${\left|\sum_{i}M_{i,i}\right|}^{2}=n^{2}L^{4}$. The denominator requires more work. We split it into two terms.

$$\underset{\begin{array}{c} i,j\\ {\vec{v}}_{i}\cdot {\vec{v}}_{j}\leq 1 \end{array}}{\sum }{\left({\vec{v}}_{i}\cdot {\vec{v}}_{j}\right)}^{2}\leq \underset{\begin{array}{c} i,j\\ {\vec{v}}_{i}\cdot {\vec{v}}_{j}\leq 1 \end{array}}{\sum }{\vec{v}}_{i}\cdot {\vec{v}}_{j}\leq cn.$$ [5]

If, for i≠j, ${\vec{v}}_{i}\cdot {\vec{v}}_{j}>1$, then (i,j) is an edge with probability 1. Thus, there can be, at most, (c−1)n such pairs. Overall, there are, at most, cn pairs such that ${\vec{v}}_{i}\cdot {\vec{v}}_{j}>1$. So, $\sum_{\begin{array}{c} i,j\\ {\vec{v}}_{i}\cdot {\vec{v}}_{j}>1 \end{array}}\left({\vec{v}}_{i}\cdot {\vec{v}}_{j}\right)\leq cnL^{4}$. Overall, we lower-bound the denominator in the rank lemma by $cn\left(L^{4}+1\right)$.

We plug these bounds into the rank lemma. We use the fact that f(x)=x/(1+x) is decreasing for positive x, and that $L\geq \sqrt{\Delta }/c$.

$$rank\left(M\right)\geq \frac{n^{2}L^{4}}{cn\left(L^{4}+1\right)}\geq \frac{n}{c}\cdot \frac{\Delta^{2}/c^{4}}{\Delta^{2}/c^{4}+1}=\frac{\Delta^{2}}{c\left(\Delta^{2}+c^{4}\right)}\cdot n.$$

### Dealing with Varying Lengths.

The math behind Eq. **4** still holds with the right approximations. Intuitively, the existence of at least Δn triangles implies that a sufficiently large number of vectors have a length of at least $\sqrt{\Delta }/c$. On the other hand, these long vectors need to be “sufficiently far away” to ensure that the vertex degrees remain low. There are many such long vectors, and they can only be far away when their dimension/rank is sufficiently high.

The rank lemma is the main technical tool that formalizes this intuition. When vectors are of varying length, the primary obstacle is the presence of extremely long vectors that create triangles. The numerator in the rank lemma sums $M_{i,i}$, which is the length of the vectors. A small set of extremely long vectors could dominate the sum, increasing the numerator. In that case, we do not get a meaningful rank bound.

But, because the vectors inhabit low-dimensional space, the long vectors from different clusters interact with each other. We prove a “packing” lemma (**Lemma 5**) showing that there must be many large positive dot products among a set of extremely long vectors. Thus, many of the corresponding vertices have large degree, and triangles incident to these vertices do not contribute to low-degree triangles. Operationally, the main proof uses the packing lemma to show that there are few long vectors. These can be removed without affecting the low-degree structure. One can then perform a binning (or “rounding”) of the lengths of the remaining vectors, to implement the proof described in the above section.

## Proof of Theorem 1

For convenience, we restate the setting. Consider a set of vectors ${\vec{v}}_{1},{\vec{v}}_{2},\ldots ,{\vec{v}}_{n}\in R^{d}$, that represent the vertices of a social network. We will also use the matrix $V\in R^{d\times n}$ for these vectors, where each column is one of the ${\vec{v}}_{i}$. Abusing notation, we will use V to represent both the set of vectors and the matrix. We will refer to the vertices by the index in [n].

Let $G_{V}$ denote the following distribution of graphs over the vertex set [n]. For each index pair i,j, independently insert (undirected) edge (i,j) with probability $\max\left(0,\min\left({\vec{v}}_{i}\cdot {\vec{v}}_{j},1\right)\right)$.

### The Basic Tools.

We now state some results that will be used in the final proof. **Lemma 2** is an existing result. For all other statements, the proofs are provided in *SI Appendix*.

**Lemma 2.** *[Rank lemma* (26)*] Consider any square matrix* $A\in R^{n\times n}$*. Then*

$${\left|\underset{i}{\sum }A_{i,i}\right|}^{2}\leq rank\left(A\right)\left(\underset{i}{\sum }\underset{j}{\sum }{\left|A_{i,j}\right|}^{2}\right).$$

**Lemma 3.** *Consider a set of s vectors* ${\vec{w}}_{1},{\vec{w}}_{2},\ldots ,{\vec{w}}_{s}$ *in* $R^{d}$.

$$\underset{\begin{array}{c} \left(i,j\right)\in \left[s\right]\times \left[s\right]\\ {\vec{w}}_{i}\cdot {\vec{w}}_{j}<0 \end{array}}{\sum }|{\vec{w}}_{i}\cdot {\vec{w}}_{j}|\leq \underset{\begin{array}{c} \left(i,j\right)\in \left[s\right]\times \left[s\right]\\ {\vec{w}}_{i}\cdot {\vec{w}}_{j}>0 \end{array}}{\sum }|{\vec{w}}_{i}\cdot {\vec{w}}_{j}|.$$

Recall that an independent set is a collection of vertices that induce no edge.

**Lemma 4.** *Any graph with* h *vertices and maximum degree* b *has an independent set of at least* h/(b+1).

**Proposition 1.** *Consider the distribution* $G_{V}$*. Let* $D_{i}$ *denote the degree of vertex* i∈[n]. $\text{E}\left[D_{i}^{2}\right]\leq \text{E}\left[D_{i}\right]+\text{E}{\left[D_{i}\right]}^{2}$.

A key component of dealing with arbitrary-length vectors is the following dot product lemma. This is inspired by results of Alon (27) and Tao (28), who get a stronger lower bound of $1/\sqrt{d}$ for *absolute values* of the dot products.

**Lemma 5.** *Consider any set of* 4d *unit vectors* ${\vec{u}}_{1},{\vec{u}}_{2},\ldots ,{\vec{u}}_{4d}$ *in* $R^{d}$*. There exists some* i≠j *such that* ${\vec{u}}_{i}\cdot {\vec{u}}_{j}\geq 1/4d$.

### The Main Argument.

We prove by contradiction. We assume that the expected number of triangles contained in the set of vertices of expected degree, at most, c is at least Δn. We remind the reader that n is the total number of vertices. For convenience, we simply remove the vectors corresponding to vertices with expected degree of at least c. Let $\hat{V}$ be the matrix of the remaining vectors, and we focus on $G_{\hat{V}}$. The expected number of triangles in $G\approx G_{\hat{V}}$ is at least Δn.

The overall proof can be thought of in three parts.

*Part 1, remove extremely long vectors:* Our final aim is to use the rank lemma (**Lemma 2**) to lower bound the rank of V. The first problem we encounter is that extremely long vectors can dominate the expressions in the rank lemma, and we do not get useful bounds. We show that the number of such long vectors is extremely small, and they can be removed without affecting too many triangles. In addition, we can also remove extremely small vectors, since they cannot participate in many triangles.

*Part 2, find a “core” of sufficiently long vectors that contains enough triangles:* The previous step gets a “cleaned” set of vectors. Now, we bucket these vectors by length. We show that there is a large bucket, with vectors that are sufficiently long, such that there are enough triangles contained in this bucket.

*Part 3, apply the rank lemma to the “core”:* We now focus on this core of vectors, where the rank lemma can be applied. At this stage, the mathematics shown in *High-Level Description of the Proof* can be carried out almost directly.

Now for the formal proof. For the sake of contradiction, we assume that $d=rank\left(\hat{V}\right)<\alpha \left(\Delta^{4}/c^{9}\right)\cdot n/{\mathrm{lg}}^{2}n$ (for some sufficiently small constant α>0).

**Part 1: Removing extremely long (and extremely short) vectors**

We begin by showing that there cannot be many long vectors in $\hat{V}$.

**Lemma 6.** *There are, at most,* 5cd *vectors of length at least* $2\sqrt{n}$.

*Proof*. Let L be the set of “long” vectors, those with a length of at least $2\sqrt{n}$. Let us prove by contradiction, and so assume there are more than 5cd long vectors. Consider a graph H=(L,E), where vectors $\vec{v_{i}},\vec{v_{j}}\in L$ (i≠j) are connected by an edge if $\frac{\vec{v_{i}}}{{\left\|{\vec{v}}_{i}\right\|}_{2}}\cdot \frac{\vec{v_{j}}}{{\left\|{\vec{v}}_{j}\right\|}_{2}}\geq 1/4n$. We choose the 1/4n bound to ensure that all edges in H are edges in G.

Formally, for any edge (i,j) in H, $\vec{v_{i}}\cdot \vec{v_{j}}\geq {\left\|{\vec{v}}_{i}\right\|}_{2}{\left\|{\vec{v}}_{j}\right\|}_{2}/4n\geq {\left(2\sqrt{n}\right)}^{2}/4n=1$. So (i,j) is an edge with probability 1 in $G\approx G_{V}$. The degree of any vertex in H is, at most, c. By **Lemma 4**, H contains an independent set I of a size of at least 5cd/(c+1)≥4d. Consider an arbitrary sequence of 4d (normalized) vectors in $I\;{\vec{u}}_{1},\ldots ,{\vec{u}}_{4d}$. Applying **Lemma 5** to this sequence, we deduce the existence of (i,j) in I (i≠j) such that $\frac{\vec{v_{i}}}{{\left\|{\vec{v}}_{i}\right\|}_{2}}\cdot \frac{\vec{v_{j}}}{{\left\|{\vec{v}}_{j}\right\|}_{2}}\geq 1/4d\geq 1/4n$. Then, the edge (i,j) should be present in H, contradicting the fact that I is an independent set. □

Denote by V′ the set of all vectors in $\hat{V}$ with length in the range $\left[n^{-2},2\sqrt{n}\right]$.

**Proposition 2.** *The expected degree of every vertex in* $G\approx G_{V\prime }$ *is, at most,* c*, and the expected number of triangles in* G *is at least* Δn/2.

*Proof*. Since removal of vectors can only decrease the degree, the expected degree of every vertex in $G_{V^{\prime }}$ is, naturally, at most, c. It remains to bound the expected number of triangles in $G\approx G_{V\prime }$. By removing vectors in V\V′, we potentially lose some triangles. Let us categorize them into those that involve at least one “long” vector (length $\geq 2\sqrt{n}$) and those that involve at least one “short” vector (length $\leq n^{-2}$) but no long vector.

We start with the first type. By **Lemma 6**, there are, at most, 5cd long vectors. For any vertex, the expected number of triangles incident to that vertex is, at most, the expected square of the degree. By **Proposition 1**, the expected degree squares is, at most, $c+c^{2}\leq 2c^{2}$. Thus, the expected total number of triangles of the first type is, at most, $5cd\times 2c^{2}\leq \Delta n/{\mathrm{lg}}^{2}n$.

Consider any triple of vectors $\left(\vec{u},\vec{v},\vec{w}\right)$ where $\vec{u}$ is short and neither of the others are long. The probability that this triple forms a triangle is, at most,

$$\begin{array}{ccc} \min\left(\vec{u}\cdot \vec{v},1\right)\cdot \min\left(\vec{u}\cdot \vec{w},1\right) & & \leq \min\left({\left\|\vec{u}\right\|}_{2}{\left\|\vec{v}\right\|}_{2},1\right)\cdot \\ \min\left({\left\|\vec{u}\right\|}_{2}{\left\|\vec{w}\right\|}_{2},1\right) & & \leq {\left(n^{-2}\cdot 2\sqrt{n}\right)}^{2}\leq 4n^{-3}. \end{array}$$

Summing over all such triples, the expected number of such triangles is, at most, 4.

Thus, the expected number of triangles in $G\approx G_{V\prime }$ is at least $\Delta n-\Delta n/{\mathrm{lg}}^{2}n-4\geq \Delta n/2$. □

**Part 2: Finding core of sufficiently long vectors with enough triangles**

For any integer r, let $V_{r}$ be the set of vectors $\left\{\vec{v}\in V\prime |{\left\|\vec{v}\right\|}_{2}\in \left[2^{r},2^{r+1}\right)\right\}$. Observe that the $V_{r}$ form a partition of V′. Since all lengths in V′ are in the range $\left[n^{-2},2\sqrt{n}\right]$, there are, at most, 3lgn nonempty $V_{r}$. Let R be the set of indices r such that $|V_{r}|\geq \left(\Delta /60c^{2}\right)\left(n/\mathrm{lg}n\right)$. Furthermore, let $V^{\prime \prime }$ be ${⋃}_{r\in R}V_{r}$.

**Proposition 3.** *The expected number of triangles in* $G\approx G_{V^{\prime \prime }}$ *is at least* Δn/8.

*Proof*. The total number of vectors in ${⋃}_{r\notin R}V_{r}$ is, at most, $3\mathrm{lg}n\times \left(\Delta /60c^{2}\right)\left(n/\mathrm{lg}n\right)\;\leq \left(\Delta /20c^{2}\right)n$. By **Proposition 1** and linearity of expectation, the expected sum of squares of degrees of all vectors in ${⋃}_{r\notin R}V_{r}$ is, at most, $\left(d+c^{2}\right)\times \left(\Delta /20c^{2}\right)n\;\leq \Delta n/10$. Since the expected number of triangles in $G\approx G_{V\prime }$ is at least Δn/2 (**Proposition 2**) and the expected number of triangles incident to vectors in $V^{\prime }\backslash V^{\prime \prime }$ is, at most, Δn/10, the expected number of triangles in $G\approx G_{V^{\prime \prime }}$ is at least Δn/2−Δn/10≥Δn/8. □

We now come to an important proposition. Because the expected number of triangles in $G\approx G_{V^{\prime \prime }}$ is large, we can prove that $V^{\prime \prime }$ must contain vectors of at least constant length.

**Proposition 4.** $\underset{r\in R}{\max}2^{r}\geq \sqrt{\Delta }/4c$.

*Proof*. Suppose not. Then every vector in $V^{\prime \prime }$ has a length of, at most, $\sqrt{\Delta }/4c$. By Cauchy–Schwartz, for every pair $\vec{u},\vec{v}\in V^{\prime \prime }$, $\vec{u}\cdot \vec{v}\leq \Delta /16c^{2}$. Let I denote the set of vector indices in $V^{\prime \prime }$ (this corresponds to the vertices in $G\approx G_{V^{\prime \prime }}$). For any two vertices i≠j∈I, let $X_{i,j}$ be the indicator random variable for edge (i,j) being present. The expected number of triangles incident to vertex i in $G\approx G_{V^{\prime \prime }}$ is

$$\text{E}\left[\underset{j\neq k\in I}{\sum }X_{i,j}X_{i,k}X_{j,k}\right]=\underset{j\neq k\in I}{\sum }\text{E}\left[X_{i,j}X_{i,k}\right]\text{E}\left[X_{j,k}\right].$$

Observe that $\text{E}\left[X_{j,k}\right]$ is, at most, $\vec{v_{j}}\cdot \vec{v_{k}}\leq \Delta /16c^{2}$. Furthermore, $\sum_{j\neq k\in I}\text{E}\left[X_{i,j}X_{i,k}\right]=\text{E}\left[D_{i}^{2}\right]$ (recall that $D_{i}$ is the degree of vertex i). By **Proposition 1**, this is, at most, $c+c^{2}\leq 2c^{2}$. The expected number of triangles in $G\approx G_{V^{\prime \prime }}$ is, at most, $n\times 2c^{2}\times \Delta /16c^{2}=\Delta n/8$. This contradicts **Proposition 3**. □

**Part 3: Applying the rank lemma to the core**

We are ready to apply the rank bound of **Lemma 2** to prove the final result. The following lemma contradicts our initial bound on the rank d, completing the proof. We will omit some details in the following proof, and provide a full proof in *SI Appendix*.

**Lemma 7.** $rank\left(V^{\prime \prime }\right)\geq \left(\alpha \Delta^{4}/c^{9}\right)n/{\mathrm{lg}}^{2}n$.

*Proof*. It is convenient to denote the index set of $V^{\prime \prime }$ be I. Let M be the Gram matrix ${\left(V^{\prime \prime }\right)}^{T}\left(V^{\prime \prime }\right)$; so, for i,j∈I, $M_{i,j}={\vec{v}}_{i}\cdot {\vec{v}}_{j}$. By **Lemma 2**, $rank\left(V^{\prime \prime }\right)=rank\left(M\right)\geq {\left(\sum_{i\in I}M_{i,i}\right)}^{2}/\sum_{i,j\in I}{\left|M_{i,j}\right|}^{2}$. Note that $M_{i,i}$ is ${\left\|\vec{v_{i}}\right\|}_{2}^{2}$, which is at least $2^{2r}$ for $\vec{v_{i}}\in V_{r}$. Let us denote $\underset{r\in R}{\max}2^{r}$ by L, so all vectors in $V^{\prime \prime }$ have a length of, at most, 2L. By Cauchy–Schwartz, all entries in M are, at most, $4L^{2}$.

We lower-bound the numerator.

$$\begin{array}{ccc} & & {\left(\underset{i\in I}{\sum }{\left\|\vec{v_{i}}\right\|}_{2}^{2}\right)}^{2}\geq {\left(\underset{r\in R}{\sum }2^{2r}|V_{r}|\right)}^{2}\geq \\ & & {\left(\underset{r\in R}{\max}2^{2r}\left(\Delta /60c^{2}\right)\left(n/\mathrm{lg}n\right)\right)}^{2}=L^{4}\left(\Delta^{2}/3600c^{4}\right)\left(n^{2}/{\mathrm{lg}}^{2}n\right). \end{array}$$

A series of technical calculations are needed to upper-bound the denominator, $\sum_{i,j\in I}{\left|M_{i,j}\right|}^{2}$. These details are provided in *SI Appendix*. The main upshot is that we can prove $\sum_{i,j\in I}{\left|M_{i,j}\right|}^{2}\leq 128cn\left(1+L^{4}\right)$.

Crucially, by **Proposition 4**, $L\geq \sqrt{\Delta }/4c$. Thus, $4^{4}c^{4}L^{4}/\Delta^{2}\geq 1$. Combining all of the bounds (and setting $\alpha <1/\left(128\cdot 3600\cdot 4^{4}\right)$),

$$\begin{array}{ccc} rank\left(V^{\prime \prime }\right) & \geq & \frac{L^{4}\left(\Delta^{2}/3600c^{4}\right)\left(n^{2}/{\mathrm{lg}}^{2}n\right)}{128cn\left(1+16L^{4}\right)}\\ & \geq & \frac{L^{4}\left(\Delta^{2}/3600c^{4}\right)\left(n/{\mathrm{lg}}^{2}n\right)}{128cn\left(4^{4}c^{4}L^{4}/\Delta^{2}+16L^{4}\right)}\geq \left(\alpha \Delta^{4}/c^{9}\right)\left(n/{\mathrm{lg}}^{2}n\right). \end{array}$$

□

## Details of Empirical Results

### Data Availability.

The datasets used are summarized in Table 1. We present here four publicly available datasets from different domains. The ca-HepPh is a coauthorship network, Facebook is a social network, and cit-HepPh is a citation network, all obtained from the SNAP graph database (29). The String_hs dataset is a protein–protein interaction network obtained from ref. 30. (The citations provide the link to obtain the corresponding datasets.)

We first describe the primary experiment, used to validate **Theorem 1** on the SVD embedding. We generated a d-dimensional embedding for various values of d using the SVD. Let G be a graph with the n×n (symmetric) adjacency matrix A, with eigendecomposition $\Psi \Lambda \Psi^{T}$. Let $\Lambda_{d}$ be the matrix with the d×d diagonal matrix with the d largest magnitude eigenvalues of A along the diagonal. Let $\Psi_{d}$ be the n×d matrix with the corresponding eigenvectors as columns. We compute the matrix $A_{d}=\Psi_{d}\Lambda_{d}\Psi_{d}^{T}$ and refer to this as the d spectral embedding of G. This is the standard principal components analysis (PCA) approach.

From the spectral embeddings, we generate a graph from $A_{d}$ by considering every pair of vertices (i,j) and generate a random value in [0,1]. If the (i,j)th entry of $A_{d}$ is greater than the random value generated, the edge is added to the graph. Otherwise, the edge is not present. This is the same as taking $A_{d}$ and setting all negative values to 0 and all values >1 to 1 and performing Bernoulli trials for each edge with the resulting probabilities. In all of the figures, this is referred to as the “SVD TDP” embedding.

### Triangle Distributions.

**Fig. 2.**
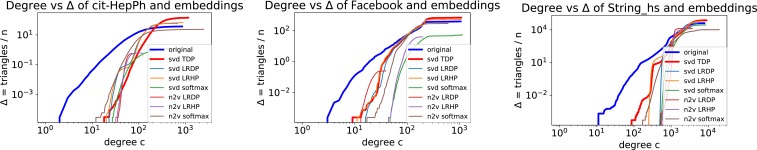
Plots of degree c vs. Δ: For each network, we plot c versus the total number of triangles only involving vertices of degree of, at most, c. We divide the latter by the number of vertices, so it corresponds to Δ, as in the main definition. In each plot, we plot these for both the original graph and the maximum Δ in a set of 100 samples from a 100-dimensional embedding. Observe how the embeddings generate graphs with very few triangles among low-degree vertices. The gap in Δ for low degree is two to three orders of magnitude in all instances.

To generate Figs. 1 and 2, we calculated the number of triangles incident to vertices of different degrees in both the original graphs and the graphs generated from the embeddings. Each of the plots shows the number of triangles in the graph on the vertical axis and the degrees of vertices on the horizontal axis. Each curve corresponds to some graph, and each point (x,y) in a given curve shows that the graph contains y triangles if we remove all vertices with a degree of at least x. We then generate 100 random samples from the 100-dimensional embedding, as given by SVD (described above). For each value of c, we plot the maximum value of Δ over all of the samples. This is to ensure that our results are not affected by statistical variation (which was quite minimal).

### Alternate Graph Models.

We consider three other functions of the dot product, to construct graph distributions from the vector embeddings. Details on parameter settings and the procedure used for the optimization are given in *SI Appendix*.

#### LRDP.

We consider the probability of an edge (i,j) to be the logistic function $L{\left(1+\exp\left(-k\left({\vec{v}}_{i}\cdot {\vec{v}}_{j}-x_{0}\right)\right)\right)}^{-1}$, where $L,k,x_{0}$ are parameters. Observe that the range of this function is [0,1], and hence can be interpreted as a probability. We tune these parameters to fit the expected number of edges, to the true number of edges. Then, we proceed as in the TDP experiment. We note that the TDP can be approximated by a logistic function, and thus the LRDP embedding is a “closer fit” to the graph than the TDP embedding.

#### *LRHP*.

This is inspired by linear models used on low-dimensional embeddings (7). Define the Hadamard product ${\vec{v}}_{i}⊙{\vec{v}}_{j}$ to be the d-dimensional vector where the rth coordinate is the product of the rth coordinates. We now fit a logistic function over linear functions of (the coordinates of) ${\vec{v}}_{i}⊙{\vec{v}}_{j}$. This is a significantly richer model than the previous model, which uses a fixed linear function (sum). Again, we tune parameters to match the number of edges.

#### Softmax.

This is inspired by low-dimensional embeddings for random walk matrices (10, 12). The idea is to make the probability of edge (i,j) proportional to softmax, $\exp\left({\vec{v}}_{i}\cdot {\vec{v}}_{j}\right)/\sum_{k\in \left[n\right]}{\vec{v}}_{i}\cdot {\vec{v}}_{k}$. This tends to push edge formation even for slightly higher dot products, and one might imagine this helps triangle formation. We set the proportionality constant separately for each vertex to ensure that the expected degree is the true degree. The probability matrix is technically undirected, but we symmetrize the matrix.

#### node2vec experiments.

We also applied node2vec, a recent deep learning-based graph embedding method (12), to generate vector representations of the vertices. We use default parameters to run node2vec. (More details are provided in *SI Appendix*.) The node2vec algorithm tries to model the random walk matrix associated with a graph, not the raw adjacency matrix. The dot products between the output vectors ${\vec{v}}_{i}\cdot {\vec{v}}_{j}$ are used to model the random walk probability of going from i to j, rather than the presence of an edge. It does not make sense to apply the TDP function to these dot products, since this will generate (in expectation) only n edges (one for each vertex). We apply the LRDP or LRHP functions, which use the node2vec vectors as inputs to a machine learning model that predicts edges.

In Figs. 1 and 2, we show results for all of the datasets. We note that, for all datasets and all embeddings, the models fail to capture the low-degree triangle behavior.

### Degree Distributions.

**Fig. 3.**
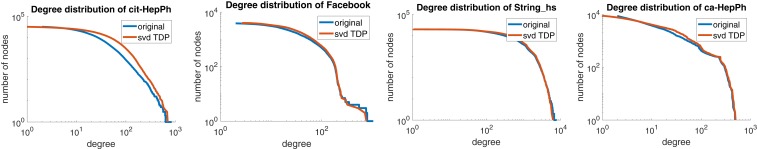
Plots of degree distributions: For each network, we plot the true degree distribution vs. the expected degree distribution of a 100-dimensional embedding. Observe how the embedding does capture the degree distribution quite accurately at all scales.

We observe that the low-dimensional embeddings obtained from SVD and TDP can capture the degree distribution accurately. In Fig. 3, we plot the degree distribution (in loglog scale) of the original graph with the expected degree distribution of the embedding. For each vertex i, we can compute its expected degree by the sum $\sum_{i}p_{ij}$, where $p_{ij}$ is the probability of the edge (i,j). In all cases, the expected degree distribution is close to the true degree distributions, even for lower degree vertices. The embedding successfully captures the “first-order” connections (degrees), but not the higher-order connections (triangles). We believe that this reinforces the need to look at the triangle structure to discover the weaknesses of low-dimensional embeddings.

## Supplementary Material

Supplementary File
