# Supplementary material for: The impossibility of low-rank representations for triangle-rich complex networks
Source: Proc Natl Acad Sci U S A. 2020 Mar 2;117(11):5631–7. doi: 10.1073/pnas.1911030117 (PMC7084093; doi:10.1073/pnas.1911030117)
Supplement: Supplementary File [file pnas.1911030117.sapp.pdf]

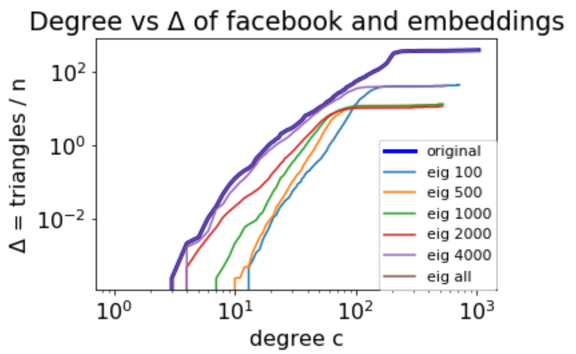

Fig. 1. Plots of degree c vs Δ, for varying rank: For the `Facebook` social network, for varying rank of embedding, we plot c versus the total number of triangles only involving vertices of degree at most c. The embedding is generated by taking the top eigenvectors. Observe how even a rank of 2000 does not suffice to match the true triangle values for low degree.

Supporting Information (SI) for "The impossibility of low rank representations for triangle-rich complex networks"

1. Further empirical details

Optimization for LRDP and LRHP: The fitting of the model was done using the Matlab function `glmfit` (Generalized Linear Model Regression Fit) (1). The distribution parameter was set to "binomial", since the total number of edges is distributed as a weighted binomial.

NODE2VEC *experimental details:* We used the optimized C++ implementation (2) for NODE2VEC, which is equivalent to the original implementation provided by the authors (3). For all our experiments, we use the default settings of walk length of 80, 10 walks per node, p=1 and q=1.

A. Detailed relationship between rank and triangle structure.

For the smallest `Facebook` graph, we were able to compute the entire set of eigenvalues. This allows us to determine how large a rank is required to recreate the low-degree triangle structure. In Figure 1, for varying rank of the embedding, we plot the corresponding triangle distribution. In this plot, we choose the embedding given by the eigendecomposition (rather than SVD), since it is guaranteed to converge to the correct triangle distribution for an n-dimensional embedding (n is the number of vertices). The SVD and eigendecomposition are mostly identical for large singular/eigenvalues, but tend to be different (up to a sign) for negative eigenvalues.

We observe that even a 1000 dimensional embedding does not capture the c vs Δ plots for low degree. Even the rank 2000 embedding is off the true values, though it is correct to within an order of magnitude. This is strong corroboration of our main theorem, which says that near linear rank is needed to match the low-degree triangle structure.

2. Full details of proof

We provide all the mathematical details that are omitted from the main body. For ease of reading, we simply provide all proof (not just omitted proofs), potentially repeating text from the main body.

For convenience, we restate the setting. Consider a set of vectors $\vec{v}_1, \vec{v}_2, \ldots, \vec{v}_n \in \mathbb{R}^d$, that represent the vertices of a social network. We will also use the matrix $V \in \mathbb{R}^{d \times n}$ for these vectors, where each column is one of the $\vec{v}_i$s. Abusing notation, we will use V to represent both the set of vectors as well as the matrix. We will refer to the vertices by the index in $[n]$.

Let $\mathcal{G}_V$ denote the following distribution of graphs over the vertex set $[n]$. For each index pair i, j, independently insert (undirected) edge (i, j) with probability $\max(0, \min(\vec{v}_i \cdot \vec{v}_j, 1))$.

A. The basic tools.

We now state some results that will be used in the final proof.

Lemma 2.1. *[Rank lemma (4)] Consider any square matrix $A \in \mathbb{R}^{n\times n}$. Then*

$$|\sum_i A_{i,i}|^2 \leq \mathrm{rank}(A)\left(\sum_i\sum_j |A_{i,j}|^2\right)$$

Lemma 2.2. *Consider a set of s vectors $\vec{w}_1, \vec{w}_2, \ldots, \vec{w}_s$ in $\mathbb{R}^d$.*

$$\sum_{\substack{(i,j)\in[s]\times[s] \\ \vec{w}_i\cdot\vec{w}_j<0}} |\vec{w}_i \cdot \vec{w}_j| \leq \sum_{\substack{(i,j)\in[s]\times[s] \\ \vec{w}_i\cdot\vec{w}_j>0}} |\vec{w}_i \cdot \vec{w}_j|$$

Proof. Note that $(\sum_{i\leq s} \vec{w}_i)\cdot(\sum_{i\leq s} \vec{w}_i) \geq 0$. Expand and rearrange to complete the proof. □

Recall that an independent set is a collection of vertices that induce no edge.

Lemma 2.3. *Any graph with h vertices and maximum degree b has an independent set of at least $h/(b+1)$.*

Proof. Intuitively, one can incrementally build an independent set, by adding one vertex to the set, and removing at most $b+1$ vertices from the graph. This process can be done at least $h/(b+1)$ times.

Formally, we prove by induction on h. First we show the base case. If $h \leq b+1$, then the statement is trivially true. (There is always an independent set of size 1.) For the induction step, let us construct an independent set of the desired size. Pick an arbitrary vertex x and add it to the independent set. Remove x and all of its neighbors. By the induction hypothesis, the remaining graph has an independent set of size at least $(h-b-1)/(b+1) = h/(b+1) - 1$. □

Claim 2.4. *Consider the distribution $\mathcal{G}_V$. Let D_i denote the degree of vertex $i \in [n]$. $\mathbf{E}[D_i^2] \leq \mathbf{E}[D_i] + \mathbf{E}[D_i]^2$.*

Proof. (of Claim 2.4) Fix any vertex $i \in [n]$. Observe that $D_i = \sum_{j\neq i} X_j$, where X_j is the indicator random variable for edge (i, j) being present. Furthermore, all the X_js are independent.

$$\begin{aligned}
\mathbf{E}[D_i^2] &= \mathbf{E}[(\sum_{j\neq i} X_j)^2] = \mathbf{E}[\sum_{j\neq i} X_j^2 + 2\sum_{j\neq j'} X_j X_{j'}] \\
&= \mathbf{E}[\sum_{j\neq i} X_j] + 2\sum_{j\neq j'} \mathbf{E}[X_j]\mathbf{E}[X_{j'}] \\
&\leq \mathbf{E}[D_i] + (\sum_{j\neq i} \mathbf{E}[X_j])^2 = \mathbf{E}[D_i] + \mathbf{E}[D_i]^2
\end{aligned}$$

□

A key component of dealing with arbitrary length vectors is the following dot product lemma. This is inspired by results of Alon (5) and Tao (6), who get a stronger lower bound of $1/\sqrt{d}$ for *absolute values* of the dot products.

Lemma 2.5. *Consider any set of $4d$ unit vectors $\vec{u}_1, \vec{u}_2, \ldots, \vec{u}_{4d}$ in $\mathbb{R}^d$. There exists some $i \neq j$ such that $\vec{u}_i \cdot \vec{u}_j \geq 1/4d$.*

DRAFT

Proof. (of Lemma 2.5) We prove by contradiction, so assume $\forall i \neq j, \vec{u}_i \cdot \vec{u}_j < 1/4d$. We partition the set $[4d] \times [4d]$ into $\mathcal{N} = \{(i,j) | \vec{u}_i \cdot \vec{u}_j < 0\}$ and $\mathcal{P} = \{(i,j) | \vec{u}_i \cdot \vec{u}_j \geq 0\}$. The proof goes by providing (inconsistent) upper and lower bounds for $\sum_{(i,j)\in\mathcal{N}} |\vec{u}_i \cdot \vec{u}_j|^2$. First, we upper bound $\sum_{(i,j)\in\mathcal{N}} |\vec{u}_i \cdot \vec{u}_j|^2$ by:

$$\begin{aligned}
&\leq \sum_{(i,j)\in\mathcal{N}} |\vec{u}_i \cdot \vec{u}_j| \qquad (\vec{u}_i\text{s are unit vectors}) \\
&\leq \sum_{i\leq 4d} \|\vec{u}_i\|_2^2 + \sum_{\substack{1\leq i\neq j\leq 4d \\ (i,j)\in\mathcal{P}}} |\vec{u}_i \cdot \vec{u}_j| \qquad (\text{Lemma 2.2}) \\
&< 4d + 16d^2/4d = 8d \quad (\text{by assumption, } \vec{u}_i \cdot \vec{u}_j < 1/4d \qquad [1]
\end{aligned}$$

For the lower bound, we invoke the rank bound of Lemma 2.1 on the $4d \times 4d$ Gram matrix M of $\vec{u}_1, \ldots, \vec{u}_{4d}$. Note that $\text{rank}(M) \leq d$, $M_{i,i} = 1$, and $M_{i,j} = \vec{u}_i \cdot \vec{u}_j$. By Lemma 2.1, $\sum_{(i,j)\in[4d]\times[4d]} |\vec{u}_i \cdot \vec{u}_j|^2 \geq (4d)^2/d = 16d$. We bound

$$\begin{aligned}
\sum_{(i,j)\in\mathcal{P}} |\vec{u}_i \cdot \vec{u}_j|^2 &= \sum_{i\leq 4d} \|\vec{u}_i\|_2^2 + \sum_{(i,j)\in\mathcal{P}, i\neq j} |\vec{u}_i \cdot \vec{u}_j|^2 \qquad [2] \\
&\leq 4d + (4d)^2/(4d)^2 \leq 5d \qquad [3]
\end{aligned}$$

Thus, $\sum_{(i,j)\in\mathcal{N}} |\vec{u}_i \cdot \vec{u}_j|^2 \geq 16d - 5d = 11d$. This contradicts the bound of Eqn. (1).

□

B. The main argument. We prove by contradiction. We assume that the expected number of triangles contained in the set of vertices of expected degree at most c, is at least Δn. We remind the reader that n is the total number of vertices. For convenience, we simply remove the vectors corresponding to vertices with expected degree at least c. Let $\hat{V}$ be the matrix of the remaining vectors, and we focus on $\mathcal{G}_{\hat{V}}$. The expected number of triangles in $G \sim \mathcal{G}_{\hat{V}}$ is at least Δn.

The overall proof can be thought of in three parts.

Part 1, remove extremely long vectors: Our final aim is to use the rank lemma (Lemma 2.1) to lower bound the rank of V. The first problem we encounter is that extremely long vectors can dominate the expressions in the rank lemma, and we do not get useful bounds. We show that the number of such long vectors is extremely small, and they can removed without affecting too many triangles. In addition, we can also remove extremely small vectors, since they cannot participate in many triangles.

Part 2, find a "core" of sufficiently long vectors that contains enough triangles: The previous step gets a "cleaned" set of vectors. Now, we bucket these vectors by length. We show that there is a large bucket, with vectors that are sufficiently long, such that there are enough triangle contained in this bucket.

Part 3, apply the rank lemma to the "core": We now focus on this core of vectors, where the rank lemma can be applied.

Now for the formal proof. For the sake of contradiction, we assume that $d = \text{rank}(\hat{V}) < \alpha(\Delta^4/c^9)\cdot n/\lg^2 n$ (for some sufficiently small constant $\alpha > 0$).

Part 1: Removing extremely long (and extremely short) vectors

We begin by showing that there cannot be many long vectors in $\hat{V}$.

Lemma 2.6. *There are at most $5cd$ vectors of length at least $2\sqrt{n}$.*

Proof. Let $\mathcal{L}$ be the set of "long" vectors, those with length at least $2\sqrt{n}$. Let us prove by contradiction, so assume there are more than $5cd$ long vectors. Consider a graph $H = (\mathcal{L}, E)$, where vectors $\vec{v}_i, \vec{v}_j \in \mathcal{L}$ $(i \neq j)$ are connected by an edge if $\frac{\vec{v}_i}{\|\vec{v}_i\|_2} \cdot \frac{\vec{v}_j}{\|\vec{v}_j\|_2} \geq 1/4n$. We choose the $1/4n$ bound to ensure that all edges in H are edges in G.

Formally, for any edge (i,j) in H, $\vec{v}_i \cdot \vec{v}_j \geq \|\vec{v}_i\|_2 \|\vec{v}_j\|_2/4n \geq (2\sqrt{n})^2/4n = 1$. So (i,j) is an edge with probability 1 in $G \sim \mathcal{G}_V$. The degree of any vertex in H is at most c. By Lemma 2.3, H contains an independent set I of size at least $5cd/(c+1) \geq 4d$. Consider an arbitrary sequence of $4d$ (normalized) vectors in I $\vec{u}_1, \ldots, \vec{u}_{4d}$. Applying Lemma 2.5 to this sequence, we deduce the existence of (i,j) in I $(i \neq j)$ such that $\frac{\vec{v}_i}{\|\vec{v}_i\|_2} \cdot \frac{\vec{v}_j}{\|\vec{v}_j\|_2} \geq 1/4d \geq 1/4n$. Then, the edge (i,j) should be present in H, contradicting the fact that I is an independent set. □

Denote by V' the set of all vectors in $\hat{V}$ with length in the range $[n^{-2}, 2\sqrt{n}]$.

Claim 2.7. *The expected degree of every vertex in $G \sim \mathcal{G}_{V'}$ is at most c, and the expected number of triangles in G is at least $\Delta n/2$.*

Proof. Since removal of vectors can only decrease the degree, the expected degree of every vertex in $\mathcal{G}_{V'}$ is naturally at most c. It remains to bound the expected number of triangles in $G \sim \mathcal{G}_{V'}$. By removing vectors in $V \setminus V'$, we potentially lose some triangles. Let us categorize them into those that involve at least one "long" vector (length $\geq 2\sqrt{n}$) and those that involve at least one "short" vector (length $\leq n^{-2}$) but no long vector.

We start with the first type. By Lemma 2.6, there are at most $5cd$ long vectors. For any vertex, the expected number of triangles incident to that vertex is at most the expected square of the degree. By Claim 2.4, the expected degree squares is at most $c + c^2 \leq 2c^2$. Thus, the expected total number of triangles of the first type is at most $5cd \times 2c^2 \leq \Delta n/\lg^2 n$.

Consider any triple of vectors $(\vec{u}, \vec{v}, \vec{w})$ where $\vec{u}$ is short and neither of the others are long. The probability that this triple forms a triangle is at most

$$\begin{aligned}
\min(\vec{u}\cdot\vec{v}, 1) \cdot \min(\vec{u}\cdot\vec{w}, 1) &\leq \min(\|\vec{u}\|_2\|\vec{v}\|_2, 1) \cdot \min(\|\vec{u}\|_2\|\vec{w}\|_2, 1) \\
&\leq (n^{-2} \cdot 2\sqrt{n})^2 \leq 4n^{-3}
\end{aligned}$$

Summing over all such triples, the expected number of such triangles is at most 4.

Thus, the expected number of triangles in $G \sim \mathcal{G}_{V'}$ is at least $\Delta n - \Delta n/\lg^2 n - 4 \geq \Delta n/2$. □

Part 2: Finding core of sufficiently long vectors with enough triangles

For any integer r, let V_r be the set of vectors $\{\vec{v} \in V' | \|\vec{v}\|_2 \in [2^r, 2^{r+1})\}$. Observe that the V_rs form a partition of V'. Since all lengths in V' are in the range $[n^{-2}, 2\sqrt{n}]$, there are at most $3\lg n$ non-empty V_rs. Let R be the set of indices r such that $|V_r| \geq (\Delta/60c^2)(n/\lg n)$. Furthermore, let V'' be $\bigcup_{r\in R} V_r$.

Claim 2.8. *The expected number of triangles in $G \sim \mathcal{G}_{V''}$ is at least $\Delta n/8$.*

Proof. The total number of vectors in $\bigcup_{r\notin R} V_r$ is at most $3\lg n \times (\Delta/60c^2)(n/\lg n) \leq (\Delta/20c^2)n$. By Claim 2.4 and linearity of expectation, the expected sum of squares of degrees of all vectors in $\bigcup_{r\notin R} V_r$ is at most $(d + c^2) \times (\Delta/20c^2)n \leq \Delta n/10$. Since the expected number of triangles in $G \sim \mathcal{G}_{V'}$ is at least $\Delta n/2$ (Claim 2.7) and the expected number of triangles incident to vectors in $V' \setminus V''$ is at most $\Delta n/10$, the expected number of triangles in $G \sim \mathcal{G}_{V''}$ is at least $\Delta n/2 - \Delta n/10 \geq \Delta n/8$. □

DRAFT

We now come to an important claim. Because the expected number of triangles in $G \sim \mathcal{G}_{V''}$ is large, we can prove that V'' must contain vectors of at least constant length.

Claim 2.9. $\max_{r \in R} 2^r \geq \sqrt{\Delta}/4c$.

Proof. Suppose not. Then every vector in V'' has length at most $\sqrt{\Delta}/4c$. By Cauchy-Schwartz, for every pair $\vec{u}, \vec{v} \in V''$, $\vec{u} \cdot \vec{v} \leq \Delta/16c^2$. Let I denote the set of vector indices in V'' (this corresponds to the vertices in $G \sim \mathcal{G}_{V''}$). For any two vertices $i \neq j \in I$, let $X_{i,j}$ be the indicator random variable for edge (i,j) being present. The expected number of triangles incident to vertex i in $G \sim \mathcal{G}_{V''}$ is

$$\mathbf{E}[\sum_{j \neq k \in I} X_{i,j} X_{i,k} X_{j,k}] = \sum_{j \neq k \in I} \mathbf{E}[X_{i,j} X_{i,k}] \mathbf{E}[X_{j,k}]$$

Observe that $\mathbf{E}[X_{j,k}]$ is at most $\vec{v_j} \cdot \vec{v_k} \leq \Delta/16c^2$. Furthermore, $\sum_{j \neq k \in I} \mathbf{E}[X_{i,j} X_{i,k}] = \mathbf{E}[D_i^2]$ (recall that D_i is the degree of vertex i.) By Claim 2.4, this is at most $c + c^2 \leq 2c^2$. The expected number of triangles in $G \sim \mathcal{G}_{V''}$ is at most $n \times 2c^2 \times \Delta/16c^2 = \Delta n/8$. This contradicts Claim 2.8. □

Part 3: Applying the rank lemma to the core

We are ready to apply the rank bound of Lemma 2.1 to prove the final result. The following lemma contradicts our initial bound on the rank d, completing the proof. We will omit some details in the following proof, and provide a full proof in the SI.

Lemma 2.10. $\mathrm{rank}(V'') \geq (\alpha\Delta^4/c^9) n/\lg^2 n$.

Proof. It is convenient to denote the index set of V'' be I. Let M be the Gram Matrix $(V'')^T(V'')$, so for $i, j \in I$, $M_{i,j} = \vec{v_i} \cdot \vec{v_j}$ By Lemma 2.1, $\mathrm{rank}(V'') = \mathrm{rank}(M) \geq (\sum_{i \in I} M_{i,i})^2 / \sum_{i,j \in I} |M_{i,j}|^2$. Note that $M_{i,i}$ is $\|\vec{v_i}\|_2^2$, which is at least 2^{2r} for $\vec{v_i} \in V_r$. Let us denote $\max_{r \in R} 2^r$ by L, so all vectors in V'' have length at most $2L$. By Cauchy-Schwartz, all entries in M are at most $4L^2$.

We lower bound the numerator.

$$\Big(\sum_{i \in I} \|\vec{v_i}\|_2^2\Big)^2 \geq \Big(\sum_{r \in R} 2^{2r} |V_r|\Big)^2$$

$$\geq \Big(\max_{r \in R} 2^{2r} (\Delta/60c^2)(n/\lg n)\Big)^2 = L^4 (\Delta^2/3600c^4)(n^2/\lg^2 n)$$

Now for the denominator. We split the sum into four parts and bound each separately.

$$\sum_{i,j \in I} |M_{i,j}|^2 = \sum_{i \in I} |M_{i,i}|^2 + \sum_{\substack{i,j \in I \\ i \neq j, M_{i,j} \in [0,1]}} |M_{i,j}|^2 + \sum_{\substack{i,j \in I \\ i \neq j, M_{i,j} > 1}} |M_{i,j}|^2 + \sum_{\substack{i,j \in I \\ M_{i,j} < 0}} |M_{i,j}|^2 \quad [4]$$

Since $|M_{i,i}| \leq L^2$, the first term is at most $4nL^4$. For $i \neq j$ and $M_{i,j} \in [0,1]$, the probability that edge (i,j) is present is precisely $M_{i,j}$. Thus, for the second term,

$$\sum_{\substack{i,j \in I \\ i \neq j, M_{i,j} \in [0,1]}} |M_{i,j}|^2 \leq \sum_{\substack{i,j \in I \\ i \neq j, M_{i,j} \in [0,1]}} M_{i,j} \leq 2cn \quad [5]$$

For the third term, we observe that when $M_{i,j} > 1$ (for $i \neq j$), then (i,j) is an edge with probability 1. There can be at most $2cn$ pairs (i,j), $i \neq j$, such that $M_{i,j} > 1$. Thus, the third term is at most $2cn \cdot (4L^2)^2 = 32cnL^4$.

Now for the fourth term. Note that M is a Gram matrix, so we can invoke Lemma 2.2 on its entries.

$$\begin{aligned}
\sum_{\substack{i,j \in I \\ M_{i,j} < 0}} |M_{i,j}|^2 &\leq L^2 \sum_{\substack{i,j \in I \\ M_{i,j} < 0}} |M_{i,j}| \\
&\leq L^2 \Big(\sum_{i \in I} |M_{i,i}| + \sum_{\substack{i,j \in I \\ M_{i,j} > 0}} |M_{i,j}|\Big) \\
&\leq 4nL^4 + L^2 \sum_{\substack{i,j \in I \\ M_{i,j} \in [0,1]}} |M_{i,j}| + 4L^4 \sum_{\substack{i,j \in I \\ M_{i,j} > 1}} 1 \\
&\leq 4nL^4 + 2cnL^2 + 8cnL^4 \quad [6]
\end{aligned}$$

Putting all the bounds together, we get that $\sum_{i,j \in I} |M_{i,j}|^2 \leq n(4L^4 + 2c + 32cL^4 + 4L^4 + 2cL^2 + 8cL^4) \leq 32n(L^4 + c(1 + L^2 + L^4))$. If $L \leq 1$, we can upper bound by $128cn$. If $L \geq 1$, we can upper bound by $128cnL^4$. In either case, $128cn(1 + L^4)$ is a valid upper bound.

Crucially, by Claim 2.9, $L \geq \sqrt{\Delta}/4c$. Thus, $4^4 c^4 L^4/\Delta^2 \geq 1$. Combining all the bounds (and setting $\alpha < 1/(128 \cdot 3600 \cdot 4^4)$),

$$\begin{aligned}
\mathrm{rank}(V'') &\geq \frac{L^4(\Delta^2/3600c^4)(n^2/\lg^2 n)}{128cn(1 + 16L^4)} \\
&\geq \frac{L^4(\Delta^2/3600c^4)(n/\lg^2 n)}{128cn(4^4c^4L^4/\Delta^2 + 16L^4)} \geq (\alpha\Delta^4/c^9)(n/\lg^2 n)
\end{aligned}$$

□

1. (2018) Matlab glmfit function (https://www.mathworks.com/help/stats/glmfit.html).
2. (2018) Node2vec c++ code (https://github.com/snap-stanford/snap/tree/master/examples/node2vec).
3. (2018) Node2vec code (https://github.com/eliorc/node2vec).
4. Swanapoel K (2014) The rank lemma (https://konradswanepoel.wordpress.com/2014/03/04/the-rank-lemma/).
5. Alon N (2003) Problems and results in extremal combinatorics, part i, discrete math. *Discrete Math* 273:31–53.
6. Tao T (2013) A cheap version of the kabatjanskii-levenstein bound for almost orthogonal vectors (https://terrytao.wordpress.com/2013/07/18/a-cheap-version-of-the-kabatjanskii-levenstein-bound-for-almost-orthogonal-vectors/).

DRAFT
